# TAK1 inhibition restores p53 expression and suppresses inflammation and hyperplasia in JIA synovial fibroblasts

**DOI:** 10.1093/rheumatology/keag186

**Published:** 2026-04-17

**Authors:** Meena A Shanta, Donavon C Lough, Lauren A Henderson, Peter A Nigrovic, Salahuddin Ahmed

**Affiliations:** Department of Pharmaceutical Sciences, Washington State University College of Pharmacy and Pharmaceutical Sciences, Spokane, WA, USA; Department of Pharmacy, East West University, Dhaka, Bangladesh; Department of Pharmaceutical Sciences, Washington State University College of Pharmacy and Pharmaceutical Sciences, Spokane, WA, USA; Division of Immunology, Boston Children’s Hospital, Harvard Medical School, Boston, MA, USA; Division of Immunology, Boston Children’s Hospital, Harvard Medical School, Boston, MA, USA; Division of Rheumatology, Inflammation, and Immunity, Brigham and Women’s Hospital, Harvard Medical School, Boston, MA, USA; Department of Pharmaceutical Sciences, Washington State University College of Pharmacy and Pharmaceutical Sciences, Spokane, WA, USA; Division of Rheumatology, University of Washington School of Medicine, Seattle, WA, USA

**Keywords:** JIA, synovial fibroblasts, TAK1, 5Z-7-oxozeaenol, synovial inflammation, p53, cytokines, CAIA, IFN-γ knock-out mice

## Abstract

**Objectives:**

This study investigated the role of TGF-β-activated kinase 1 (TAK1) in synovial inflammation and hyperplasia in JIA synovial fibroblasts (JIASFs).

**Methods:**

Patient-derived JIASFs were treated with TNF-α, IL-1β and IFN-γ with and without various TAK1 inhibitors. ELISA, Western blotting, RNA sequencing, cell proliferation and immunofluorescence were performed to study protein expression and JIASFs’ functions. *In vivo* efficacy of TAK1 inhibitors was tested in a collagen antibody-induced arthritis (CAIA) model in IFN-γ knock-out mice.

**Results:**

JIASFs exhibited elevated TAK1 and reduced basal p53 expression compared with human foreskin fibroblasts. IL-1β-activated TAK1 suppressed p53, promoting inflammatory marker expression. TAK1 inhibitors 5Z-7-oxozeaenol or 5Z (IC_50_: 22.8 nM) and NG-25 (IC_50_: 492 nM) effectively blocked IL-1β-induced TAK1 activation, reducing inflammation. Notably, 5Z selectively restored IL-1β-suppressed p53 and p21 levels and demonstrated anti-proliferative effects via cell proliferation assays and reduced PCNA expression. Among tested inhibitors, 5Z was most effective in suppressing the combined pro-inflammatory effects of IL-1β, TNF-α and IFN-γ, reducing COX-2, VCAM-1, cadherin-1, IL-6, IL-8, CXCL5 and MMP-3 expression by 80%. Furthermore, 5Z restored p53 expression more efficiently than MAPK or NF-κB pathway inhibitors. RNA sequencing confirmed its anti-inflammatory and anti-proliferative properties in JIASFs. *In vivo*, daily intraperitoneal administration of 5Z (2 mg/kg) from day 3 significantly ameliorated collagen antibody-induced arthritis (CAIA) in IFN-γ knock-out mice, highlighting its therapeutic potential.

**Conclusion:**

TAK1 inhibition restores p53 functions in JIASFs and downregulates synovial inflammation, which warrants further studies that target TAK1 to treat JIA.

Rheumatology key messages

- The differential expression of TAK1 and p53 in JIASFs compared to foreskin fibroblasts indicates a pathogenic phenotype.
- Inhibition of TAK1 limits pro-inflammatory phenotypes of JIASFs by suppressing IL-1β-mediated inflammation and cell proliferation *in vitro*.
- The *in vivo* studies also supported the anti-inflammatory potential of TAK1 inhibition in chronic arthritis.

## Introduction

JIA encompasses several forms of chronic inflammatory arthritis in children, with a female predilection [1,2]. While the aetiology of JIA remains elusive, genetics and environmental factors are known to trigger the activation of autoreactive immune cells and their subsequent infiltration into the synovium, causing chronic and persistent inflammation [1, 3,4]. The sequelae of chronic pain limit the movement of the joints, interrupt regular activities and diminish the overall quality of life through progressive joint damage [5,6]. Although the introduction of cytokine-targeted therapies has improved the disease outcome, many patients remain partially or non-responsive, and most patients who achieve remission experience frequent disease flares if medications are discontinued [7–11]. The limitations of the current therapies and the economic and psychological burdens of the disease urge more research to shift from highly targeted therapies to a multitarget approach [6, 12].

TGF-β-activated kinase 1 (TAK1) is a mammalian serine/threonine kinase belonging to the mitogen-activated kinase kinase kinase (MAP3K) family. Being central to signal transduction of a wide array of stimuli like TGF-β, TNF-α, IL-1β, bone morphogenetic proteins (BMPs) and Toll-like receptor 4 (TLR4) ligands, TAK1 is a critical determinant of cell fate [13–16]. Following the binding of the ligands to their respective receptors, an alteration to their transmembrane domain initiates a signalling cascade leading to posttranslational modification-associated activation of intracellular proteins (IRAK1/4 or TRAF2/6). Such activation facilitates the formation of the TAK1/TAB-1 complex and the autophosphorylation of TAK1. The autophosphorylation-induced activation of TAK1 further phosphorylates downstream mitogen-activated protein kinases (MAPKs) and the nuclear factor κB (NF-κB) pathway, propelling the nuclear translocation of key inflammatory transcription factors NF-κBp65 and activation protein 1 (AP-1) that regulate cell proliferation, differentiation, apoptosis and inflammation [17]. While knockdown of TAK1 in the germline is embryonically lethal, conditional knockdown in mouse embryonic fibroblasts impaired TNF-α- and IL-1β-induced cytokine production. Cell-specific deletion of TAK1 in B cells and dendritic cells impaired the activation of both cell types [18,19]. Similarly, T cell-specific deletion resulted in impaired thymocyte development, including Treg cell differentiation, survival and T cell activation [20]. Inhibition of TAK1 also impaired mast cell degranulation [21]. Given its key involvement in regulating immune cells, the therapeutic potential of TAK1 was evaluated in several autoimmune conditions [17, 22].

Although the involvement of several immune cells in JIA is well characterized, the participation of synovial fibroblasts (SFs) in JIA pathogenesis is still scarce. SFs are highly enriched in JIA synovial tissues and are responsible for producing the strongest outgoing signals to recruit adaptive immune cells in inflamed microenvironments [23]. Such findings necessitate therapeutic interventions to limit fibroblasts’ contribution to arthritis induction in JIA. The synovial microenvironments of JIA are enriched with numerous pro-inflammatory cytokines, including TNF-α and IL-1β [4, 24–26]. The pleiotropic roles of these cytokines in autoimmunity led to the introduction of several biologics to target them in the JIA treatment schedule, and hence, anti-TNF and anti-IL-1β therapy are considered the first-line biological therapies in both non-systemic and systemic JIA, respectively [27–29]. Given its critical position downstream of TNF-α and IL-1β, the highest TAK1 expression in cultured fibroblasts (according to the Genotype-Tissue Expression portal), and the prominent abundance of SFs in JIA synovial tissue to recruit cells of adaptive immunity, we investigated the efficacy and mechanism of targeting TAK1 in JIASFs.

## Methods

### Reagents and antibodies

The details of the reagents and experimental methods are provided in Supplementary Data S1.

### Treatment of JIASFs

The procurement, maintenance and characterization of human fibroblast cells are given in Supplementary Data S1. The procurement and use of SFs were conducted under the protocols approved by the Washington State University IRB (#19170) and guidelines of the Helsinki Declaration.

Human JIASFs were treated with IL-1β (0.1–10 ng/ml) and TNF-α (0.1–20 ng/ml) for 24 h to study the expression of inflammatory markers. To understand the effect of cytokines on TAK1 activation, JIASFs were treated with IL-1β (10 ng/ml) and TNF-α (20 ng/ml) up to 2 h. To investigate the impact of TAK1 inhibition on signalling pathways and inflammatory markers, JIASFs were treated with TAK1 inhibitors- 5Z-7-oxozeaneol (5Z), NG-25, HS-276 or takinib for 2 h, followed by IL-1β (10 ng/ml) stimulation for 15 min and 24 h, respectively. For the cytokines cocktail experiment, JIASFs were pre-treated with 5Z (0.25 µM), NG-25 (1 µM), HS-276 (10 µM) and takinib (10 µM) for 2 h, followed by cytokines cocktail [IL-1β (1 ng/ml), TNF-α (20 ng/ml) and IFN-γ (5 ng/ml)] treatment for 24 h or 15–30 min. For the inhibitors experiment, JIASFs were pre-treated with NF-κB*i* (200 µM), ERK*i* (10 µM), JNK*i* (10 µM), p38*i* (10 µM) and 5Z (0.25 µM) for 2 h prior to IL-1β (10 ng/ml) stimulation for 24 h. Upon completion of the experiments, cell lysates are prepared in RIPA buffer (50 mM Tris pH 7.6, 150 mM NaCl, 1 mM EDTA, 1% Triton X-100, 0.1% SDS, 0.5% deoxycholate and one tablet each for protease inhibitor and Phos-Stop by Roche per 10 ml) and further processed for Western blotting. Cell supernatants from 24 h experiments were also collected and processed for ELISA. The process of nuclear fraction preparation was described previously [30].

### Collagen antibody-induced arthritis in IFN-γ knock-out mice

We used the collagen antibody-induced arthritis (CAIA) instead of CFA to induce arthritis in IFN-γ knock-out mice, as C57BL/6 background mice show resistance to developing arthritis induced by CFA alone [31]. In addition, the role of TNF-α and IL-1β in driving arthritis induction in this model, and the pleiotropic roles of these cytokines in JIA pathogenesis [25, 29, 32], provided a rationale to use CAIA to induce arthritis. The animal experiment protocol is approved by the IACUC of Washington State University (protocol #6359). Briefly, thirteen 6-weeks-old female mice were purchased from the Jackson Laboratory (Cat. B6.129S7-*Ifng^tm1Ts/J^/^J^*) and were acclimated for 7 days. The mice were divided into three groups: naïve (*n* = 3), CAIA (*n* = 5) and CAIA + 5Z (*n* = 5). On the day of the experiment (day 0), both the CAIA and CAIA + 5Z groups received CAIA mAb cocktail (1.5 mg/ml, intraperitoneally (i.p.), Chondrex, Inc. Cat. 53010). At day 3, these mice received 25 µg LPS (i.p., Chondrex, Inc. Cat. 53010). The CAIA + 5Z group received 5Z (2 mg/kg), intraperitoneally daily from day 03 to day 10. The Naïve and CAIA group received appropriate vehicle control. The body weight, ankle circumference and articular index were measured and calculated as described previously [33].

### Statistical analysis

GraphPad Prism (GraphPad Software Corporation, San Diego, CA, USA) was used to analyse the results. Statistical analysis was performed using the one-way analysis of variance test, followed by Dunnett’s multiple comparison test or Tukey’s multiple comparison test and Student’s independent *t*-test. *P < 0.05, **P < 0.01 and ***P < 0.001 were considered statistically significant. Values were represented as mean ± standard error mean (SEM).

## Results

### TAK1 is overexpressed in JIASFs and plays an important role in cytokine signalling

**Figure 1:**
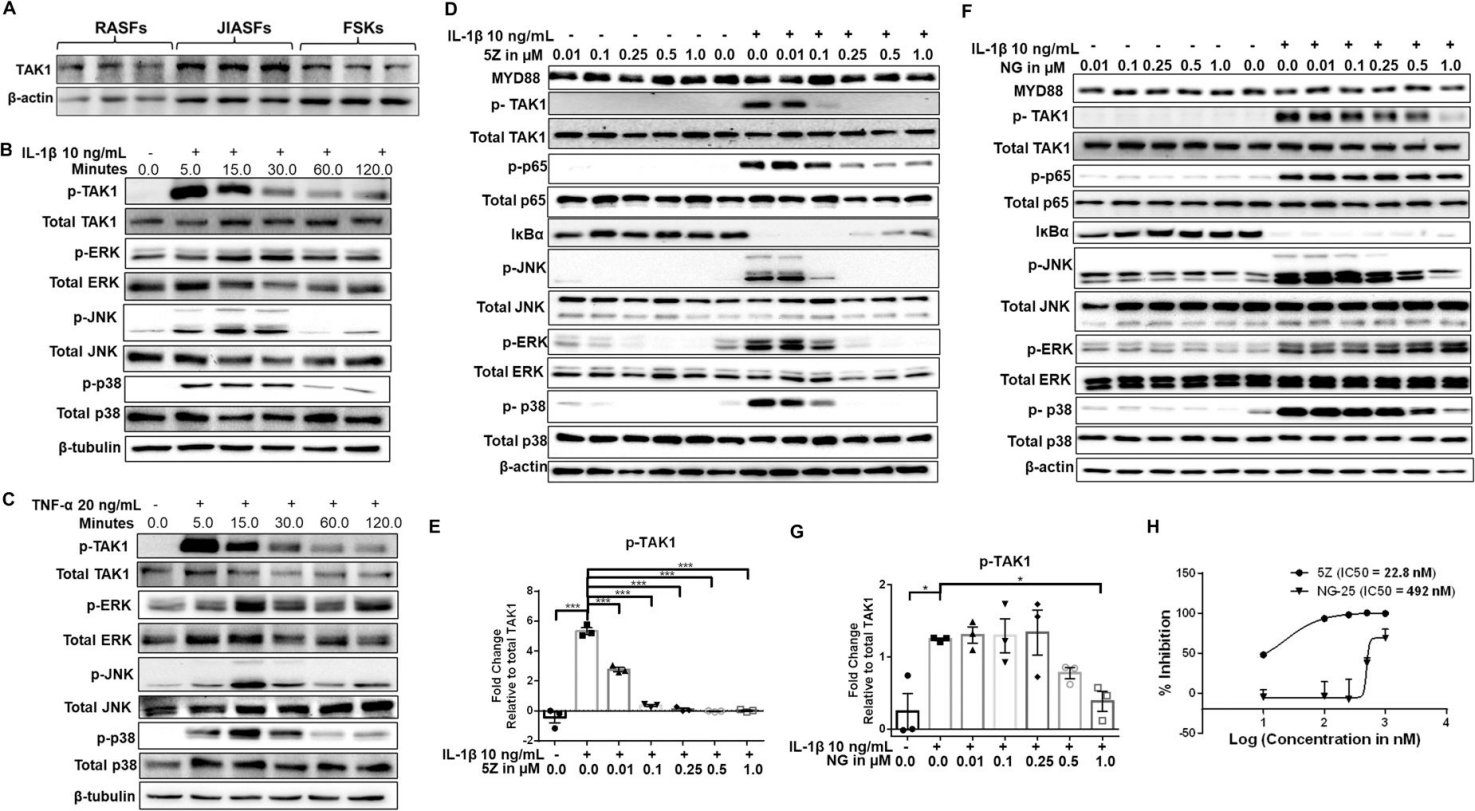
Aberrant expression of TAK1 requires inhibition of TAK1 in JIASFs to suppress downstream cytokine signalling. (**A**) JIASFs had elevated basal expression of TAK1 compared with RASF and FSKs (*n* = 5). The results were analysed using the one-way ANOVA, followed by Tukey’s multiple comparison test. (**B & C**) The time dependent activation of TAK1 and MAPKs in JIASFs following IL-1β and TNF-α stimulation. (**D & F**) 5Z and NG (NG-25) suppressed the activation of TAK1 and its downstream targets of NF-κB and MAPK pathways in a dose-dependent manner. (**E & G**) The densitometric analysis of inhibition of TAK1 phosphorylation by 5Z and NG-25, respectively. (**H**) The IC_50_ curve of 5Z and NG-25. The results were analysed using the one-way ANOVA, followed by Dunnett’s test. *P < 0.05, **P < 0.01 and ***P < 0.001 were considered statistically significant, *n* = 3 (1 × 10^5^ cells/well in 6-well plates). The values were represented as mean ± SEM, compared with IL-1β treatment

Western blot analysis of basal TAK1 expression was significantly higher in JIASFs compared with foreskin fibroblasts (FSKs) and similar to the expression in RA synovial fibroblasts (RASFs) (Fig. 1A and Supplementary Fig. S1B; *P* < 0.05). We studied the temporal expression of TAK1 activation and found that IL-1β (10 ng/ml) or TNF-α (20 ng/ml) induced p-TAK1^Thr184/187^ within 5 min of stimulation and sustained it up to 120 min (Fig. 1B and C). TAK1 activation triggered downstream phosphorylation of MAPKs (p-ERK, p-p38 and p-JNK) that peaks between 15 and 30 min by both the cytokines (Fig. 1B and C and S1C-D; *P* < 0.05, *P* < 0.01 and *P* < 0.001). This activation correlated with a dose-dependent production of IL-6 and IL-8 in cytokine-activated JIASFs (Supplementary Fig. S1E–H).

The elevated expression of TAK1 led us to study the impact of TAK1 inhibition using an irreversible covalent kinase inhibitor (5Z), and three noncovalent kinase inhibitors—NG-25, HS-276 and takinib in IL-1β-mediated activation of JIASFs. Our study revealed that 5Z inhibited the activation of p-TAK1 (∼90% inhibition at dose 0.1 µM of 5Z, *P* < 0.001) with an IC_50_ of 22.8 nM and its downstream effectors of MAPK pathways, p-JNK (∼88% inhibition at 0.1 µM, *P* < 0.01 and 0.001), p-ERK (∼90% inhibition at dose 0.5 µM) and p-p38 (∼99% inhibition at 0.25 µM, *P* < 0.001) in a dose-dependent manner (Fig. 1D, E, and H & Supplementary Fig. S2A). We also observed a ∼50% inhibition of phosphorylation of p65, which was further evident by the inhibition of degradation of I-κBα at 0.25 µM of 5Z. Being a reversible inhibitor of TAK1, NG-25 also inhibited the phosphorylation of TAK1 by ∼70% at 1 µM concentration (IC_50_ 492 nM; *P* < 0.05). A significant inhibition of phosphorylation of JNK and p38 was also observed by NG-25 (Fig. 1F–H & Supplementary Fig. S2B). By contrast, recently discovered TAK1 inhibitors HS-276 and takinib enhanced IL-1β-induced p-TAK1 expression (Supplementary Fig. S3A and B), an observation similar to our recently published work [34].

### RNA sequencing analysis reveals the gene signatures altered by TAK1 inhibition in IL-1β-activated JIASFs

**Figure 2:**
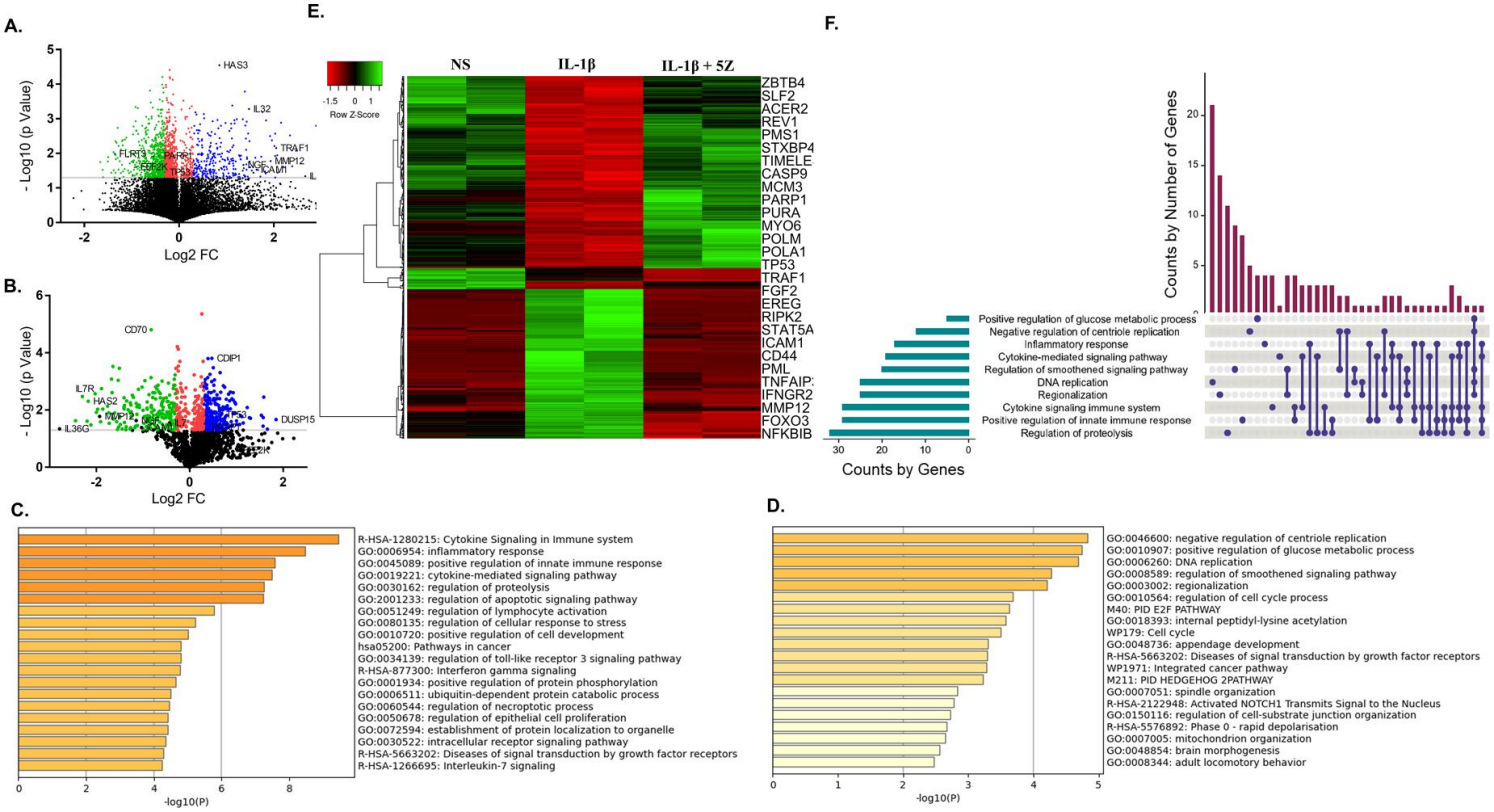
RNA sequencing revealed the 5Z-mediated reversal of cytokine-driven inflammatory and hyperproliferative properties of JIASFs. The volcano plots depicted the magnitude of alteration of genes between non-stimulated and IL-1β-stimulated groups (**A**) and between IL-1β only and IL-1β with 5Z groups (**B**). The colored dots above the grey line represented DEGs altered significantly, and the blue and green dots represented DEGs having a fold change ≥2 and *P* < 0.05. The gene ontology study showed the pathways altered due to 5Z-mediated suppression of genes (**C**) and upregulation of genes (**D**). The heatmap (**E**) reflected the gene expression profiles in different treatment groups. (**F**) The upset plot showed the distribution of genes in the top five pathways upregulated and downregulated by 5Z

Compared with non-stimulated JIASFs, ∼19 000 DEGs were altered (Fig. 2A), and ∼2000 DEGs were significantly altered by IL-1β (*P* < 0.05) and the effect of TAK1 inhibition by 5Z was investigated on those DEGs (Fig. 2B). Our results showed that ∼700 genes were significantly modulated by 5Z, of which ∼250 genes were ≥2-fold upregulated and ∼200 genes were ≥2-fold downregulated when compared with IL-1β-treated controls (Fig. 2B). The heatmap in Fig. 2C represented the magnitude of altered expression of IL-1β activated DEGs. Gene ontology (GO) conducted on those ∼450 genes revealed that 5Z suppressed the genes involved in ‘*cytokine signalling in the immune system*’, ‘*inflammatory response*’, ‘*positive regulation of innate immune response*’, ‘*cytokine mediated signalling pathways’*, etc. More importantly, 5Z restored JIASFs’ cellular homeostasis by upregulating the genes involved in the ‘*negative regulation of centriole replication*’, ‘*regulation of cell cycle process*’, ‘*E2F pathway*’ and ‘*cell cycle*’ (Fig. 2D and E). Some of the most notable upregulated genes were *TP53, PARP1, ICAM1 and NFKBIB*. Later, we investigated the distribution and overlapping of the genes involved in the top five upregulated and downregulated pathways, revealed by the GO study (Fig. 2F). *RIPK2* and *KAT2A* were the only DEGs shared by all five enriched pathways, modulated by 5Z.

### TAK1 inhibition suppressed the expression of IL-1β-induced inflammatory markers

**Figure 3:**
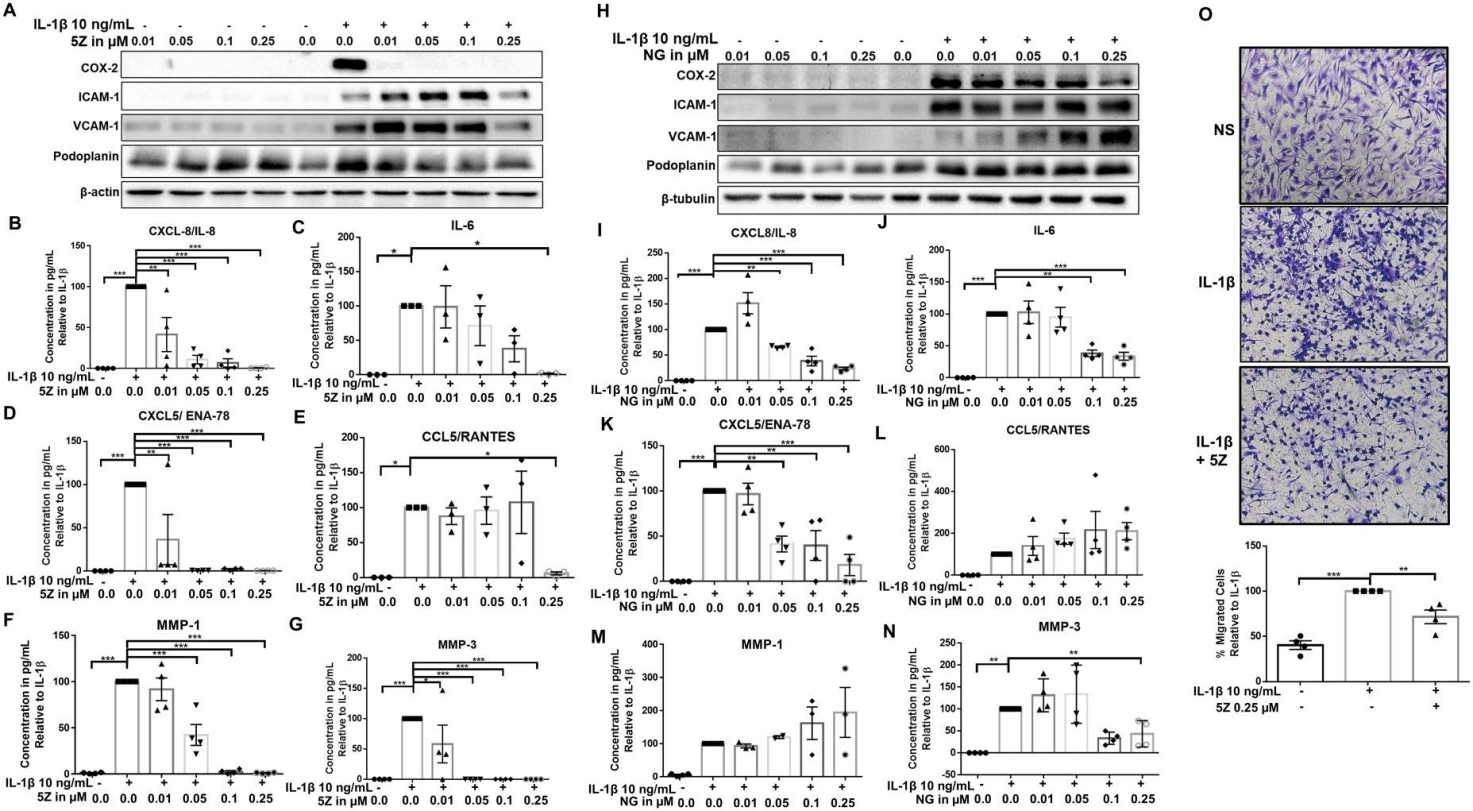
Inhibition of TAK1 suppressed the cytokine-driven expression of inflammatory markers in JIASFs. (**A**–**N**) Pre-treatment with 5Z and NG-25 suppressed the IL-1β-driven expression of cellular and secretory soluble inflammatory mediators by JIASFs. (**H**) The trans-well migration assay revealed the potential of 5Z in limiting JIASFs’ migratory potential (2.5 × 10^4^ cells/insert). The results were analysed using one-way ANOVA, followed by Dunnett’s test. *P < 0.05, **P < 0.01 and ***P < 0.001 were considered statistically significant, *n* = 3 or 4 (1 × 10^5^ cells/well in 6-well plates). The values were represented as mean ± SEM, compared with IL-1β treatment

To study the prospect of TAK1 inhibition, JIASFs were treated with 5Z and NG-25 with and without IL-1β treatment for 24 h, and the expression of pro-inflammatory markers was measured. The expression of COX-2 (*P* < 0.001), ICAM-1, VCAM-1 (*P* < 0.01) and podoplanin (*P* < 0.01) was markedly enhanced by IL-1β (Fig. 3A and Supplementary Fig. S4A & B). Inhibition of TAK1 with 5Z significantly reduced the expression of COX-2 by ∼90% at 0.05 µM and VCAM-1 by ∼60% at 0.25 µM. The maximum inhibition for ICAM-1 (∼29%) and podoplanin (∼28%) was achieved at 0.25 µM of 5Z (Fig. 3A and Supplementary Fig. S4). Additionally, we tested the conditioned media for the production of MMPs, chemokines, and cytokines in the IL-1β and 5Z-treated groups. Inhibition of TAK1 with 5Z significantly inhibited the expression of MMP-1 (*P* < 0.001), MMP-3 (*P* < 0.001), IL-6 (*P* < 0.05), CXCL8/IL-8 (*P* < 0.01 & 0.001), CCL5/RANTES (*P* < 0.05) and CXCL5/ENA-78 (*P* < 0.01 & 0.001) (Fig. 3B–G). In comparison, NG-25 inhibited the expression only of COX-2 production by JIASFs (*P* < 0.05 & 0.01), and the production of CXCL8/IL-8 (*P* < 0.01 & 0.001), IL-6 (*P* < 0.01 & 0.001), CXCL5/ENA-78 (*P* < 0.01 & 0.001) and MMP-3 (*P* < 0.01) in IL-1β-activated JIASFs (Fig. 3H–N and Supplementary Fig. S4B). The suppression of chemokines and adhesion molecules by 5Z led us to conduct a trans-well migration assay, where our findings showed that 5Z significantly prevented the IL-1β-induced migration of JIASFs *in vitro* (Fig. 3O).

### TAK1 inhibition restored cellular homeostasis by enhancing p53 expression in JIASFs

**Figure 4:**
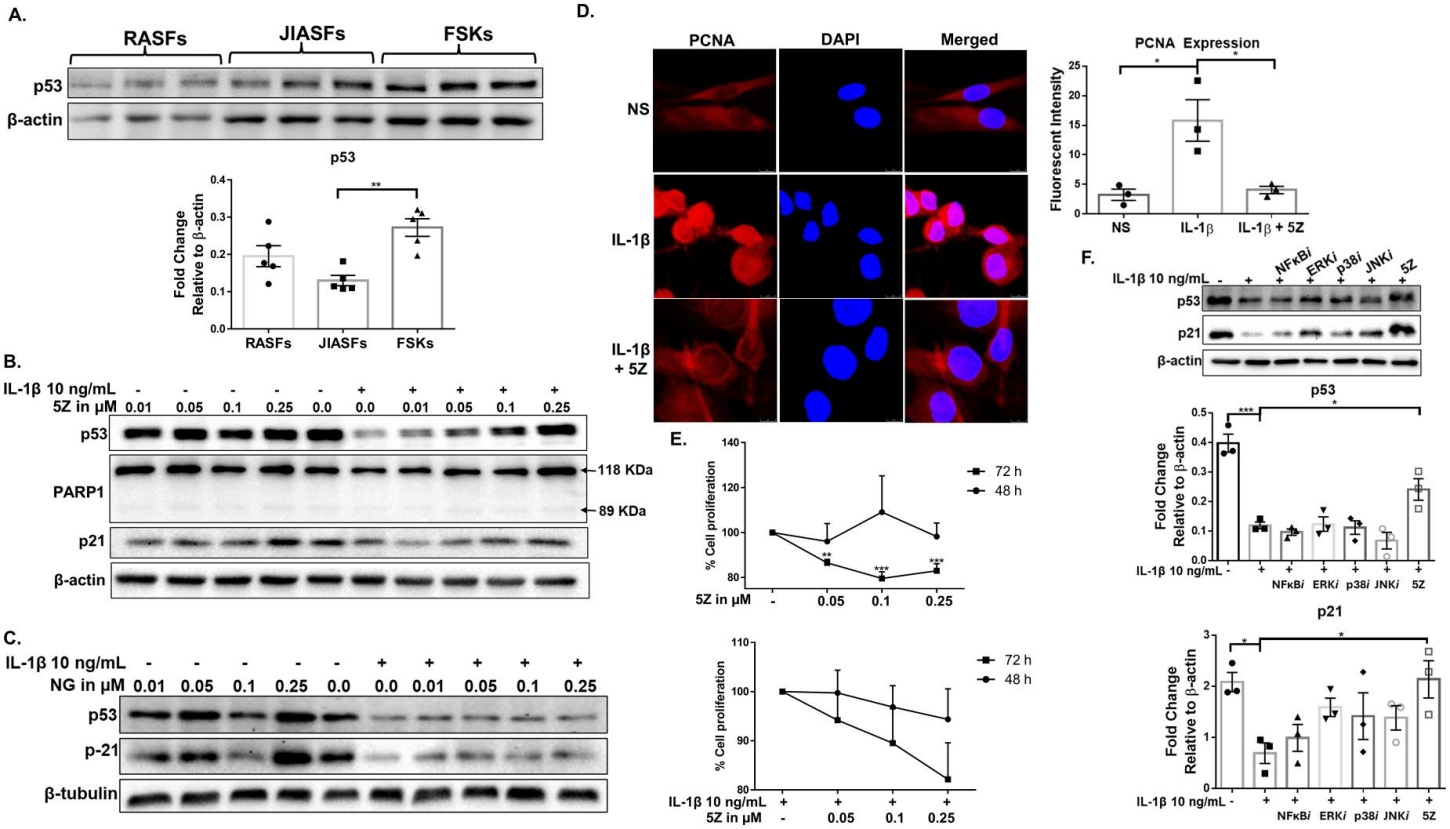
Inhibition of TAK1 limits IL-1β-induced hyperproliferation of JIASFs through the p53–p21 axis. (**A**) The basal expression of p53 was measured and compared between RASFs and FSKs (*n* = 5). The results were analysed using the one-way ANOVA, followed by Tukey’s multiple comparison test. **P < 0.01 was considered statistically significant. (**B & C**) JIASFs were treated with IL-1β with and without 5Z, and NG (NG-25) pre-treatment, and the expression of p53 and its downstream targets was measured. (**D**) The expression of PCNA was measured via immunofluorescence (2.0 × 10^4^ cells/chamber). (**E**) The CyQUANT cell proliferation assays revealed the anti-proliferative potential of 5Z in IL-1β-mediated JIASFs’ proliferation (1.0 × 10^4^ cells/well of 96-well plate). (**F**) JIASFs were pre-treated with inhibitors of TAK1, NF-κB and MAPK pathways, followed by IL-1β stimulation for 24 h, and the expression of p53 and p21 was measured. The results were analysed using the one-way ANOVA, followed by Dunnett’s test. *P < 0.05, **P < 0.01 and ***P < 0.001 were considered statistically significant, *n* = 3 or 4 (1 × 10^5^ cells/well for the rest of the experiments). The values were represented as mean ± SEM, compared with IL-1β treatment

The GO findings led us to investigate the role of p53 in JIASFs’ proliferation. JIASFs expressed a significantly low level of p53 when compared with FSKs (Fig. 4A; *P* < 0.01), which was further reduced by ∼70% upon IL-1β activation (Fig. 4B and Supplementary Fig. S5A; *P* < 0.01). Interestingly, 5Z maintained the basal p53 levels and showed a remarkable dose-dependent restoration of p53 expression in the presence of IL-1β in JIASFs (Fig. 4B and Supplementary Fig. S5A; *P* < 0.05). This result prompted us to determine the impact on the downstream targets of p53. First, the inhibition of TAK1 had no marked effect on the apoptotic proteins, such as PARP1, Bak, Mcl-1 and MDM2 (Fig. 4B and Supplementary Fig. S5B). However, the expression of p21, an immediate downstream target of p53, was enhanced by ∼44% with 5Z treatment compared with IL-1β-treated controls (*P* < 0.05). However, we did not observe any upregulation of p53 and p21 in NG-25-treated JIASFs (Fig. 4C). Given the role of p21 in suppressing cell proliferation, we also measured the expression of PCNA—a marker of cell proliferation. In line with our previous finding, 5Z at 0.25 µM significantly reduced PCNA expression by ∼75% (Fig. 4D). The cell proliferation assay further confirmed the hyperproliferative role of IL-1β and the efficacy of 5Z to control JIASFs’ proliferation via TAK1 inhibition (Fig. 4E; *P* < 0.001). To determine whether pharmacologically targeting TAK1 with 5Z is superior to the inhibition of individual MAPKs or the NF-κB pathway, we pre-treated JIASFs with individual MAPK and NF-κB inhibitors or 5Z, followed by IL-1β stimulation. Western blot analysis showed that only 5Z was capable of fully restoring p53 and p21, which were suppressed by IL-1β (Fig. 4F; *P* < 0.05).

### Inhibition of TAK1 dampened the pro-inflammatory phenotype of JIASFs

**Figure 5:**
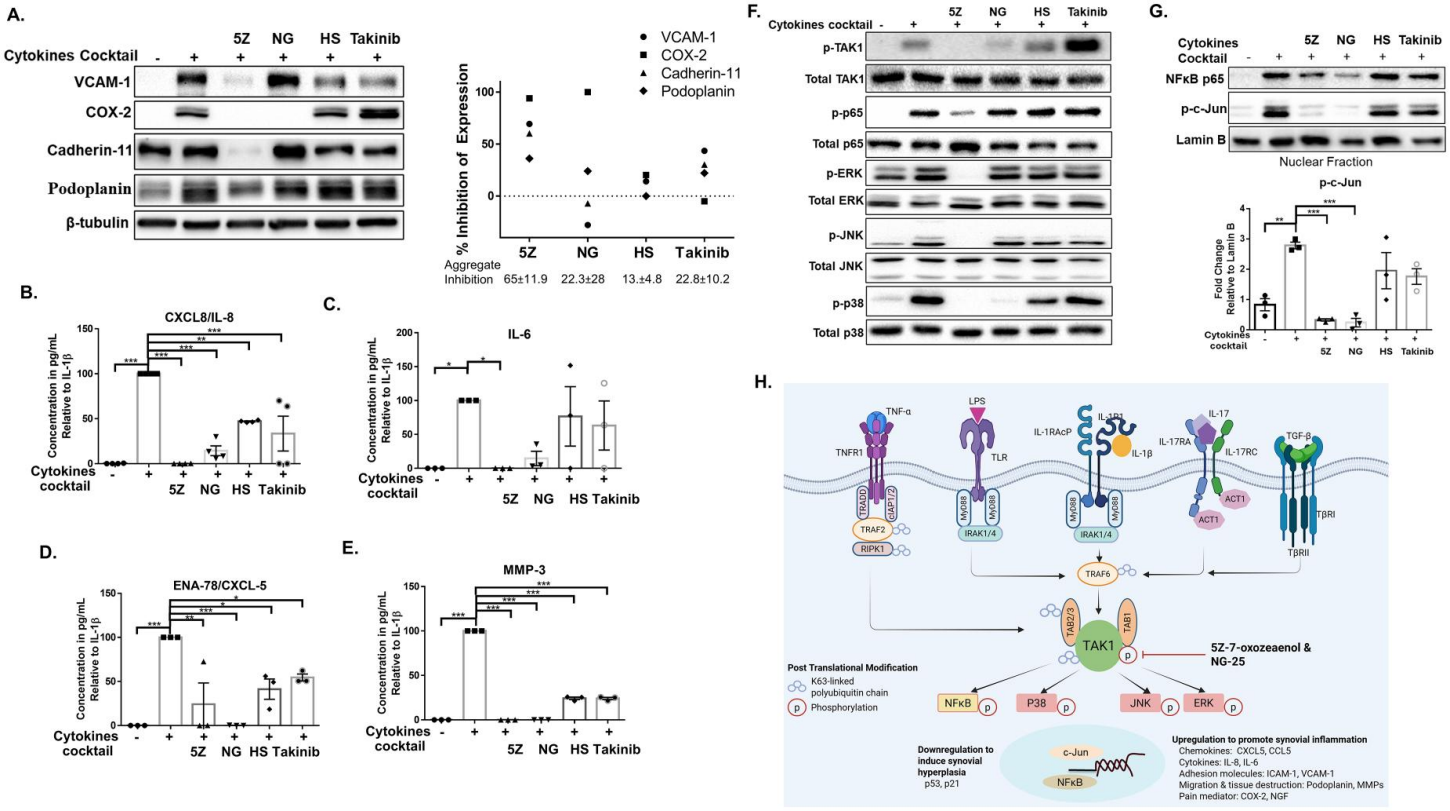
5Z suppressed the malignant transformation of JIASFs in a cytokine-enriched pro-inflammatory condition. (**A–E**) The JIASFs were pre-treated with 5Z, NG, HS-276 and takinib, followed by stimulation with a cytokine cocktail containing IFN-γ, TNF-α and IL-1β for 24 h. The expression of inflammatory markers was measured and compared between different TAK1 inhibitors. JIASFs were treated with a cytokine cocktail for 15 min with and without prior treatment with different TAK1 inhibitors to measure the phosphorylation of TAK1 and its downstream targets (**F**) and 30 min to measure the extent of translocation of p-c-Jun and NFκB p65 in the nuclear fraction (**G**). (**H**) The schematic diagram represented the critical involvement of TAK1 in the signalling pathways of multiple cytokines (created with https://BioRender.com/wxay9o4). The results were analysed using one-way ANOVA, followed by Dunnett’s test. *P < 0.05, **P < 0.01 and ***P < 0.001 were considered statistically significant, *n* = 3 or 4 (1 × 10^5^ cells/well). The values were represented as mean ± SEM, compared with cytokine cocktail treatment

IL-1β, TNF-α and IFN-γ are collectively responsible for driving the aggressive SFs phenotype. Smith *et al.* reported that stimulation with a triple combination of IL-1β (1 ng/ml), TNF-α (20 ng/ml) and IFN-γ (5 ng/ml) resulted in chromatin-accessible sites enriched for AP-1 family motifs, similar to those of activated lining fibroblasts isolated *ex vivo.* Such enrichment of AP-1 family motifs is known to confer a tissue-destructive phenotype in SFs, which correlates with disease activity in adult RA [35,36]. This compelled us to investigate the potential of TAK1 inhibition in the presence of this cytokine combination (IL-1β/TNF-α/IFN-γ). Our findings show that among all the tested inhibitors, 5Z produced the most significant aggregate suppression of inflammatory markers [5Z (80%) > NG-25 (58%) > HS-276 (33%) > takinib (40%)] (Fig. 5A and Supplementary Fig. S6A). Individually, 5Z inhibited the cellular expression of COX-2 by 94% (*P* < 0.05), cadherin-11 by 60% (*P* < 0.05) and VCAM-1 by 70% (*P* < 0.05). 5Z also downregulated podoplanin expression by 36%. Although NG-25 failed to suppress the expression of VCAM-1 and cadherin-11 but inhibited the expression of COX-2 (by 100%, *P* < 0.05) and podoplanin (by 24%). Although not significant, HS-276 and takinib at 10 µM were able to downregulate the expression of VCAM-1 (by 15% and 44%) and cadherin-11 (by 20% and 30%), respectively. Additionally, HS-276 suppressed the expression of COX-2 by 20%, and takinib inhibited podoplanin by 22% (Fig. 5A). A significant inhibition of IL-8, ENA-78 and MMP-3 expression was achieved by different TAK1 inhibitors, with 5Z and NG-25 having the most potent effect, while HS-276 and takinib failed to inhibit cytokine-induced IL-6 production in JIASFs (Fig. 5B–E & Supplementary Fig. S6).

Investigating the impact of all four TAK1 inhibitors on signalling pathways activated by cytokine combination, our results showed that 5Z was the most effective in suppressing the phosphorylation of TAK1 and its downstream targets (Fig. 5F). A significant inhibition of p-ERK by 100% (*P* < 0.001), p-JNK (100% *P* < 0.05) and p-p38 by 98% (*P* < 0.01) was observed in the 5Z-treated group. NG-25 also suppressed the phosphorylation of TAK1 by 68% and p38 by 97% (*P* < 0.001), respectively. The presence of IFN-γ is reported to enhance the efficacy of HS-276 [37], which might have contributed to a surprising reduction of phosphorylation of TAK1 by 26% in the HS-276-treated group (Fig. 5F and Supplementary Fig. S6B). We also observed a slight reduction in the phosphorylation of ERK, p38 and JNK, while the takinib group enhanced the phosphorylation of TAK1 and its downstream targets in the presence of pro-inflammatory cytokines (Fig. 5F). We isolated the nuclear extract and measured the translocation of p-c-Jun and NF-κB p65–transcription factors of the NF-κB and MAPKs pathway. In consistence with our signalling data, 5Z and NG-25 suppressed the translocation p-c-Jun by ∼88% and ∼90% (*P* < 0.001). Both 5Z and NG-25 also suppressed the translocation of NF-κB p65 by ∼40% (Fig. 5G and Supplementary Fig. S6C). The schematic diagram overall represents the involvement of TAK1 in multiple signalling pathways and the impact of 5Z and NG-25, resulting in the reduction of inflammatory genes (5H).

### 5Z ameliorated collagen antibody-induced arthritis in IFN-γ knock-out mice

**Figure 6:**
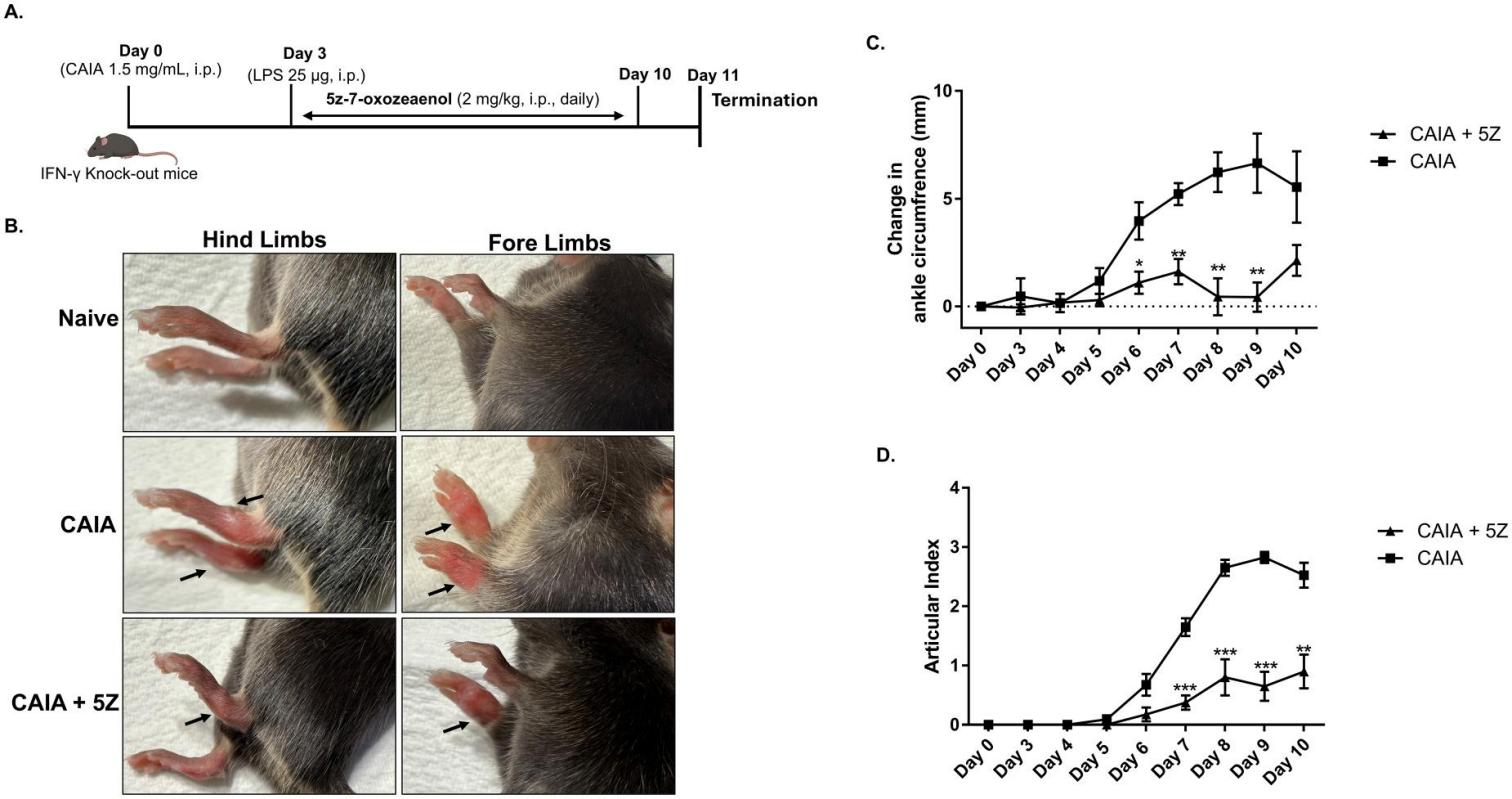
5Z inhibited the incidence of arthritis in IFN-γ knock-out mice. (**A**) Experimental protocol detailing the study to elucidate the potential of 5Z in suppressing synovial inflammation (partially created with https://BioRender.com/gtcirni). (**B**) The representative image of mice fore and hind limbs at day 10. (**C** & **D**) The change in ankle circumference and articular index demonstrated the anti-inflammatory potential of 5Z in IFN-γ knock-out mice. The results were analysed using the Student’s independent *t*-test. *P < 0.05, **P < 0.01 and ***P < 0.001 were considered statistically significant. The values were represented as mean ± SEM, compared with the CAIA group

The arthritis in IFN-γ knock-out mice reportedly resembles several clinical phenotypes of a subgroup of JIA and has been used to study its clinical pathology [38,39]. The profound effect of 5Z in suppressing IL-1β-induced inflammation in JIASFs prompted us to include 5Z only in this *in vivo* study. The CAIA group began to show signs of arthritis around day 6, which peaked by day 9, showing ∼55% increase in ankle circumference compared with the naïve group (*P* < 0.01) (Fig. 6C and D). Compared with the CAIA group on day 9, the 5Z treatment significantly reduced the change in ankle circumference by 93% (*P* < 0.01) and the articular index by 72% (*P* < 0.001) when compared with the control CAIA group (Fig. 6B–D).

## Discussion

Targeting cytokines has been a valuable yet not fully effective approach to treat JIA, which demands a shift in focus on identifying non-immunosuppressive small-molecule signalling inhibitors that can potentially limit multiple cytokine-driven synovitis in JIA. This is the first study to report the prospect of targeting TAK1 to impede the multiple pro-inflammatory cytokine-driven transformation of JIASFs. Our RNA sequencing data indicated that suppression of TAK1 inhibits IL-1β-driven inflammatory and proliferative pathways in JIASFs. The *in vitro* experiments also corroborated our RNA sequencing data, which showed suppression of inflammatory markers and p53 upregulation to counteract the pro-inflammatory effects of cytokines. The significant suppression of arthritis induction and severity *in vivo*, also validated *in vitro* findings. Thus, our findings suggest that inhibiting TAK1 is an effective therapeutic approach to restore cellular homeostasis in JIASFs and limit their contribution to cytokine-driven synovial inflammation.

JIASFs remain the major source of adhesion molecules, inflammatory chemokines and matrix-degrading MMPs in the synovial microenvironment [24,25, 40]. Synovitis is a hallmark of aggressive synovial inflammation, triggered by infiltrating immune cells that activate fibroblast-mediated tissue destruction [1]. This study provided evidence of a significantly lower expression of p53 in JIASFs compared with FSKs, which was further suppressed by IL-1β. IL-1β is known to induce cell proliferation in RA, osteoarthritic and dermal fibroblasts [41]. Consistent with our findings, Schauer *et al.* also reported that IL-1β is a negative regulator of p53 in normal ovarian fibroblasts [42]. However, targeting p53 with drugs has remained a challenge [38], and managing inflammation without treating synovial hyperplasia is also not clinically beneficial. Wellenstein *et al.* found that p53 null cancer cells facilitate bone marrow-derived macrophages to release IL-1β by secreting WNT ligands [39]. Moreover, the isolated SFs from p53 null mice possessed twice the cell proliferation capacity, marked by severe synovial hyperplasia, tissue destruction and elevated expression of IL-1β and IL-6 [43]. A similar protective role of p53 against arthritis induction is also reported in the adjuvant-induced arthritis model in rats. While the study showed an already known association between p53 and cytokine-induced SFs mediated inflammation through NF-κB and MAPK pathways, it fell short of providing an underlying molecular mechanism or a target that can be utilized to regulate complex signalling pathways [44]. Despite the availability of information regarding the involvement of IL-1β and p53 in inflammation and synovial hyperplasia, the impact of currently recommended bDMARDs in managing synovial hyperplasia and restoring p53 expression levels is scarce. Matsumura *et al.* reported that etanercept does not have any effect on controlling RASFs proliferation [45]. Histopathologic analysis of the synovium of RA patients showed that etanercept failed to limit vascular and synovial lining proliferation when compared with methotrexate and infliximab [46,47]. Thus, failure to limit the synovial hyperplasia by most of the current therapies encouraged the introduction of a cyclin-dependent kinase inhibitor, Seliciclib, in phase 1b clinical trial as an adjunct therapy in RA [48]. Such findings signify the prospects of TAK1 inhibition as a potential approach to restoration of the p53–p21 axis to suppress synovial hyperplasia and inflammation.

The overactivation of p53 facilitates neuronal death associated with ischaemia, stroke and Alzheimer’s disease. Restoration of p53 expression with MDM2 inhibitors remains successful in certain types of cancer. However, patients often experience thrombocytopenia and neutropenia requiring withdrawal of the drugs. Overexpression of p53 in mice conferred cancer resistance and premature ageing. It is widely believed that, to induce cell death, p53 expression must cross a threshold, and restoration of p53 to the cellular level induces cell cycle arrest, especially in cell types such as endothelial cells and fibroblasts [49,50] further validating our findings.

The Genotype-Tissue Expression (GTEx) portal (https://www.gtexportal.org/home/gene/MAP3K7) substantiates the higher expression of TAK1 in fibroblasts compared with lymphocytes, conferring these cells with the highest relevance to study the role of TAK1 function and targeted therapies. However, more comprehensive studies, such as organ-on-chip systems that permit a complex model enriched with SFs and other immune cells like macrophages, B cells or T cells, would enhance the impact of the findings. Nonetheless, this study provides an in-depth analysis of signal transduction pathways in JIASFs and the role of TAK1 in p53 regulation and inflammation, and, thus providing a strong rationale for further testing of such treatment for JIA.

## Supplementary Material

keag186_Supplementary_Data

## Data Availability

The data will be available upon request.
