# Supplementary material for: TAK1 inhibition restores p53 expression and suppresses inflammation and hyperplasia in JIA synovial fibroblasts
Source: Rheumatology (Oxford). 2026 Apr 17;65(4):keag186. doi: 10.1093/rheumatology/keag186 (PMC13127760; doi:10.1093/rheumatology/keag186)
Supplement: keag186_Supplementary_Data [file keag186_supplementary_data.docx]

**METHODS**

**Reagents and Antibodies**

Recombinant human IL‐1β, TNF-α, and IFN-γ were procured from R&D Systems (Minneapolis, MN, Cat. #201-LB, 210-TA, and 285-IF-100, respectively). Antibodies for β-actin, GAPDH, BAK, PCNA, Podoplanin, Total TAK1, IκBα, p53, Lamin B, and MDM2 were purchased from Santa Cruz Biotech (Santa Cruz, CA, Cat. sc-47778, sc-47724, sc-832, sc-56, sc-376695, sc-166562, sc-371, sc-126, sc-374015, and sc-13161, respectively). Antibodies for MCL1, PARP1, MYD88, p-p65^Ser536^, NF-κB-p65, p-p38^Thr180/Tyr182^, p38 MAPK, SAPK/JNK^Thr183/Tyr185^, SAPK/JNK, p-TAK1^Thr184/187^, p-Erk(1/2)^Thr202/Tyr204^, p44/42 MAPK (Erk1/2), Thy1/CD90, FAP-α, p21, p-c-Jun^Ser73^, HRP-linked anti-rabbit IgG and HRP-linked anti-mouse IgG were purchased from Cell Signaling Technology (Danvers, MA, Cat. #5453, #9542, #4283, #3033, #8242, #4511, #8690, #9251, #9252, #4508, #4370, #4695s, #13801, #66562, #2947s, #9164s, #7074 and #7076, respectively). Human anti-ICAM1/CD54, anti-VCAM1(CD106) and HRP-linked anti-goat IgG antibodies were procured from R&D Systems (Minneapolis, MN, Cat. BBA17, BBA19, and HAF017 respectively). COX-2 (mouse) polyclonal antibody was purchased from Cayman Chemical (Ann Arbor, MI, Cat. aa 584-598). Alexa Fluor™ 594 donkey anti-mouse IgG secondary antibody (Eugene, OR, Cat. #A21203) was purchased from Invitrogen™. 5Z-7-oxozeaenol (Cat. 17459), and NG-25 (Cat. 27579), were purchased from Cayman Chemical (Ann Arbor, MI). Takinib (Cat. SML2216, Ammonium pyrrolidine di-thio-carbamate (NF-κB inhibitor or NF-κB*i*), PD98059 (ERK inhibitor or ERK*i*), SB203580 (p38 inhibitor or p38*i*), and SP600125 (JNK inhibitor or JNK*i*) were purchased from Millipore/Sigma (Burlington, MA). HS-276 (HS, Cat. 144141) was purchased from MedChem Express (Monmouth Junction, NJ).

**Isolation and culturing of cells**

JIASFs were isolated from the synovial fluids of de-identified JIA patients following protocols mentioned elsewhere [1, 2]. The details of the JIASFs and Rheumatoid arthritis synovial fibroblasts (RASFs) donors are provided in *Supplementary Table 1*. The procurement and use of SFs were conducted under the protocols approved by the Institutional Review Board (IRB) and guidelines of the Helsinki Declaration. Foreskin fibroblasts (FSKs) were purchased from the Coriell Institute for Medical Research (Camden, NJ). RASFs were isolated as described earlier [3]. The JIASFs, RASFs, and FSKs were cultured in RPMI 1640 medium supplemented with 10% fetal bovine serum (FBS), 5000 U/ml penicillin, 5 mg/ml streptomycin, and 10 µg/ml gentamicin and were maintained in a humidified atmosphere (37°C, 5% CO_2_). Isolated JIASFs were propagated to passage 4 to ensure pure fibroblast lineage (Supplementary Fig. S1A). All the cells used in the experiments were between passages 4 and 10.

**Western immunoblotting**

Equal amounts of protein were resolved on 7.5-12% SDS-PAGE gels in reducing conditions using Western blotting protocols described earlier [4]. The densitometric analysis was performed using Image Lab 6.1 software (Bio-Rad Laboratories, Hercules, CA).

**Determination of soluble inflammatory markers secreted from JIASFs via enzyme-linked immunosorbent assay (ELISA)**

The conditioned media was analyzed to quantitate MMP-1 (Cat. DY901B-05), MMP-3 (Cat. DY513-05), IL-6 (Cat. DY206), IL-8/CXCL8 (Cat. DY208), RANTES/CCL5 (Cat. DY278-05), and ENA-78/CXCL5 (Cat. DY254) production using Duoset ELISA kits from R&D Systems (Minneapolis, MN) following the manufacturer’s instructions. Results were analyzed using MicroPlate Manager Software version 6.3 (California, USA).

**RNA sequencing and bioinformatic analysis**

JIASFs were treated with IL-1β with and without 5Z pretreatment, and the RNA was extracted using RNeasy Mini Kit (Cat. 74104, QIAGEN, Hilden, Germany) following the manufacturer's instructions. The poly-T oligo-attached magnetic beads were utilized for mRNA purification, and following adaptor ligation, purified mRNA was used for cDNA library preparation. The library was then sequenced on the Illumina platform (Novogene, San Diego, CA). The raw data were processed and mapped to the reference genome using the fastp and Hisat2 v2.0.5 software, respectively. The FPKM (Fragments Per Kilobase of transcript sequence per Million) values were generated for each gene. The differentially expressed genes (DEGs) were calculated by comparing the FPKM values between the two groups. Two-tailed Student’s t-test was utilized to determine the statistical significance. A volcano plot and heatmap were generated using GraphPad Prism 6 (San Diego, CA) and Heatmapper (<http://www.heatmapper.ca/expression/>), respectively. Genes having fold change ≥2 and p<0.05 were considered to conduct Gene Ontology (GO) using Metascape (https://metascape.org/gp/index.html#/main/step1).

**Cell Migration Assays**

Approximately 2.5 × 10^4^ cells were seeded into the Transwell® chambers (8.0 μm Transparent PET Membrane, Corning, Cat. No. 353097) and placed in a well of the 24-well plate filled with regular media. On the day of the experiment, the cells were stimulated with IL-1β (10 ng/mL) for 48 h, with and without 2 h pretreatment with 5Z. After 24 h, the cells at the bottom layer of the inserts were stained with 0.1% crystal violet for 30 min [5]. The inserts were then washed with PBS to remove the excess stain and imaged with the Leica DFC450 C system. The number of cells migrated was counted using Image J 1.54g (NIH, USA).

**Immunofluorescence**

To detect the expression of PCNA, 2 x 10^4^ JIASFs were seeded in a Falcon 8-chamber polystyrene-treated slide (Cat. 53106-306, Avantor, PA, USA). For the experiment, the cells were incubated with IL-1β for 24 h with and without 2 h pretreatment with 5Z (0.25 µM). After 24 h, cells were fixed in 4% paraformaldehyde for 15 min, followed by permeabilization with 0.1% Triton-X for 10 min. The cells were then incubated with the primary antibody overnight, following the blocking of cells in 1% BSA for 1 h. The next day, the cells were incubated with the secondary antibody for 1 h. The nuclei were then stained with 4,6-diamidino-2-phenylindole (DAPI), and the images were taken with a Leica DMi8 confocal microscope, and the fluorescent intensity was measured using Image J 1.54g (USA).

**Cell proliferation assays**

To validate the impact of TAK1 inhibition in suppressing cell proliferation, CyQUANT® Direct Cell Proliferation Assay Kit (Cat. C35011, Thermo Fisher, Waltham, MA) was utilized. Briefly, 1 × 10^4^ cells were seeded in a 96-well plate. At confluency, the cells were serum-starved for 2 h and treated with IL-1β up to 72 h, with and without 2 h pretreatment with 5Z. After the experiment, 100 μL of CyQUANT GR dye/lysis buffer (prepared following the manufacturer’s instructions) was added to each well and incubated for 6 min. The absorbance was taken at 485/535 nm [5].

**Supplementary Figures**

**
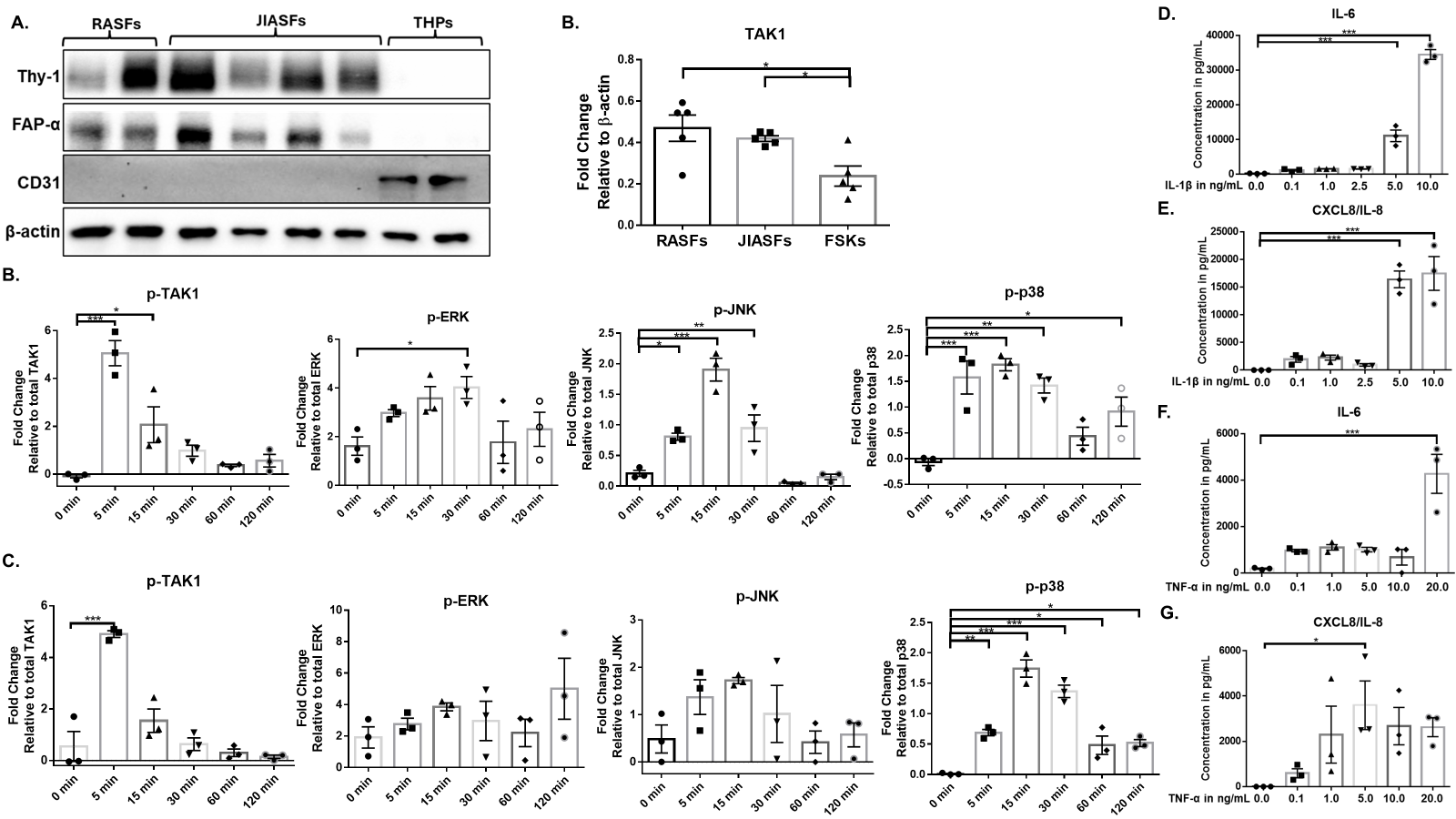
**

**Fig. S1: A.** Characterization of JIASFs’ fibroblast lineage**. B-C.** Densitometric analysis of Western blots mentioned in Figure 1 A-C. **D-G.** JIASFs were treated with TNF-α and IL-1β for 24 h, and the expression of IL-6 and CXCL-8/IL-8 was measured through ELISA. The results were analysed using One-way ANOVA followed by Dunnett’s test and Tukey's multiple comparison test. *p<0.05, **p<0.01, and ***p<0.001 were considered statistically significant. The values were represented as mean ± SEM, compared with IL-1β treatment.


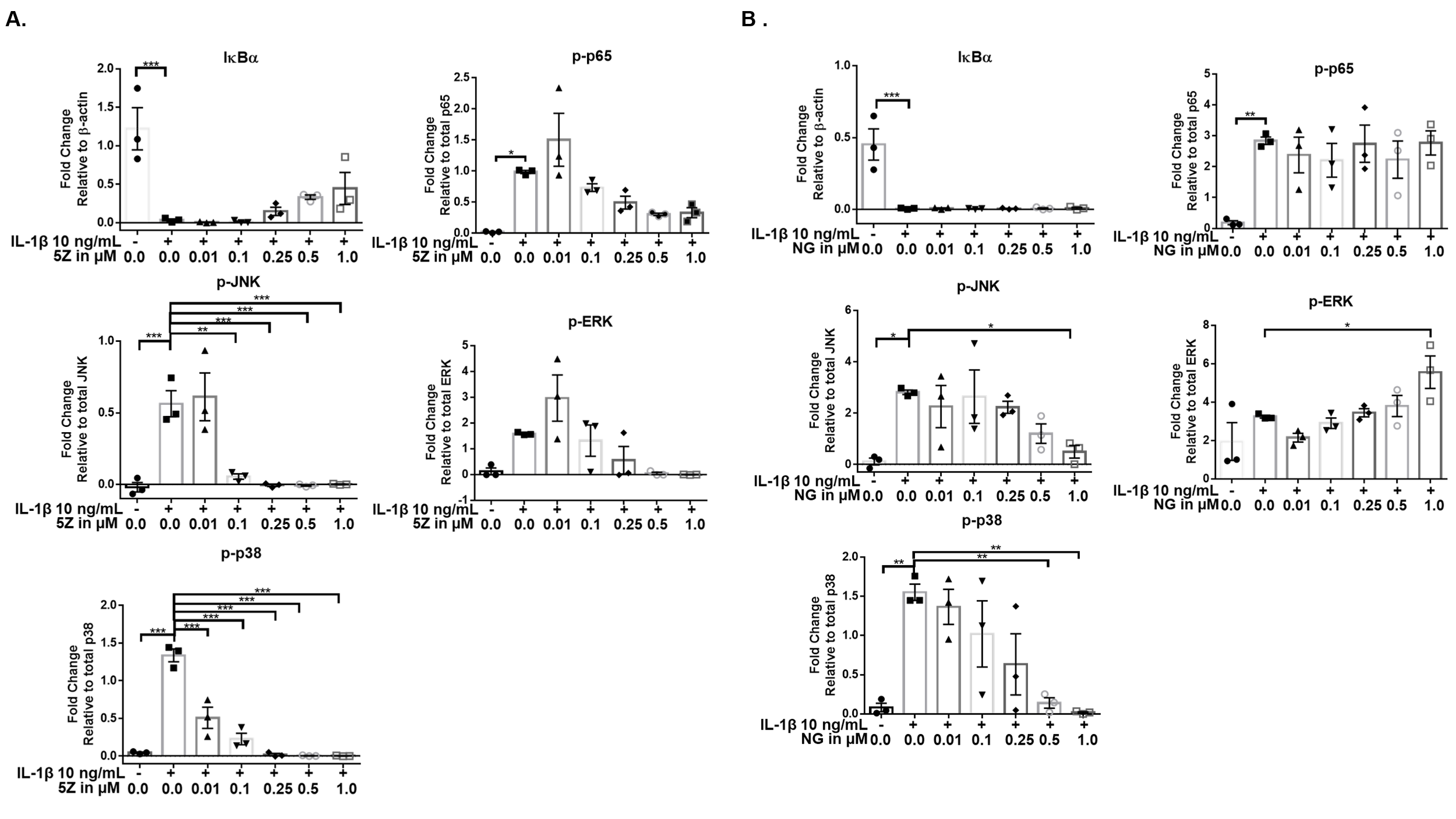


**Fig. S2:** Densitometric analysis of Western blots mentioned in Figure 1D & F. The results were analyzed using One-way ANOVA followed by Dunnett’s test. *p<0.05, **p<0.01 and ***p<0.001 were considered statistically significant. The values were represented as mean ± SEM, compared with IL-1β treatment.


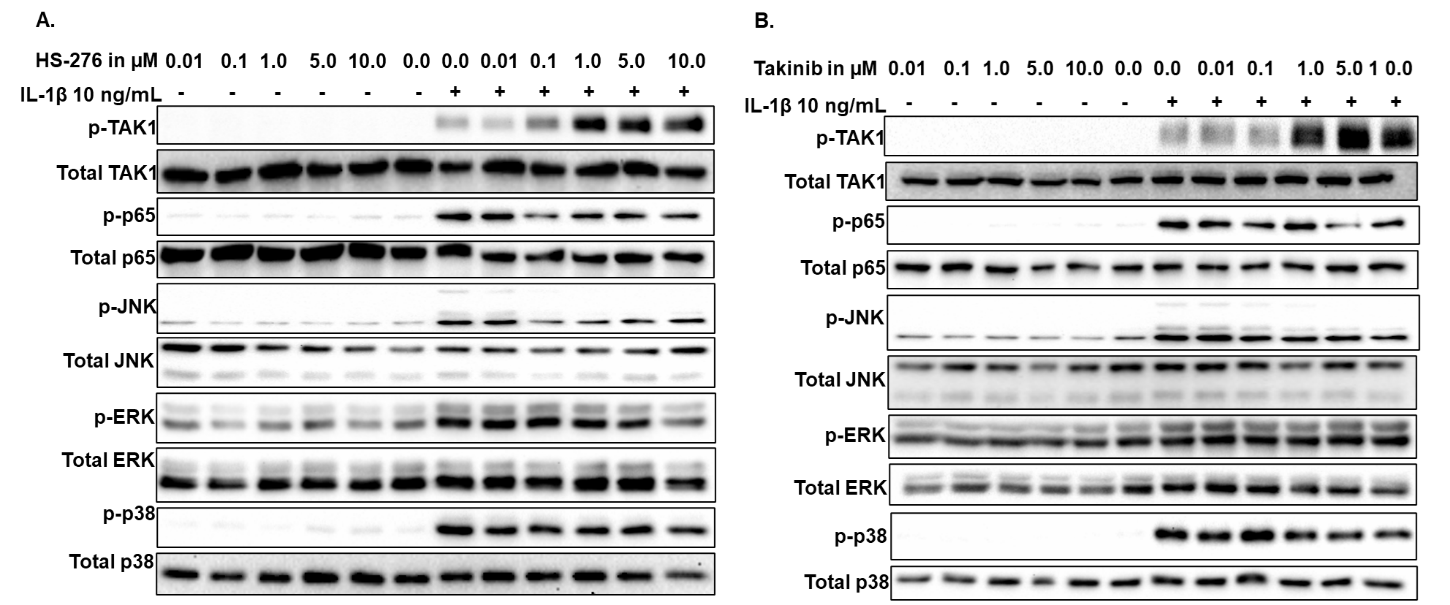


**Fig. S3:** JIASFs were pretreated with HS-276 and takinib for 2 h followed by IL-1β stimulation for 15 min, and the expression of pTAK1 and its downstream targets was measured.


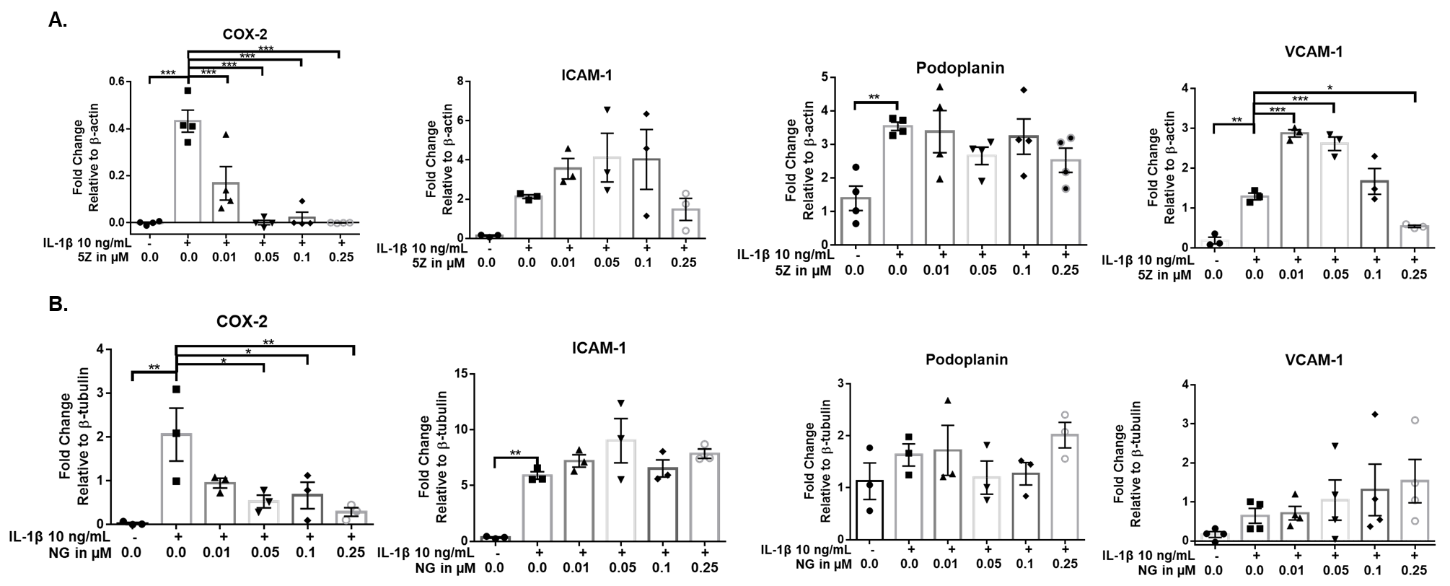


**Fig. S4: A-B.** Densitometric analysis of Western blots mentioned in Figure 3A & H. The results were analyzed using One-way ANOVA followed by Dunnett’s test. *p<0.05, **p<0.01 and ***p<0.001 were considered statistically significant. The values were represented as mean ± SEM, compared with IL-1β treatment.


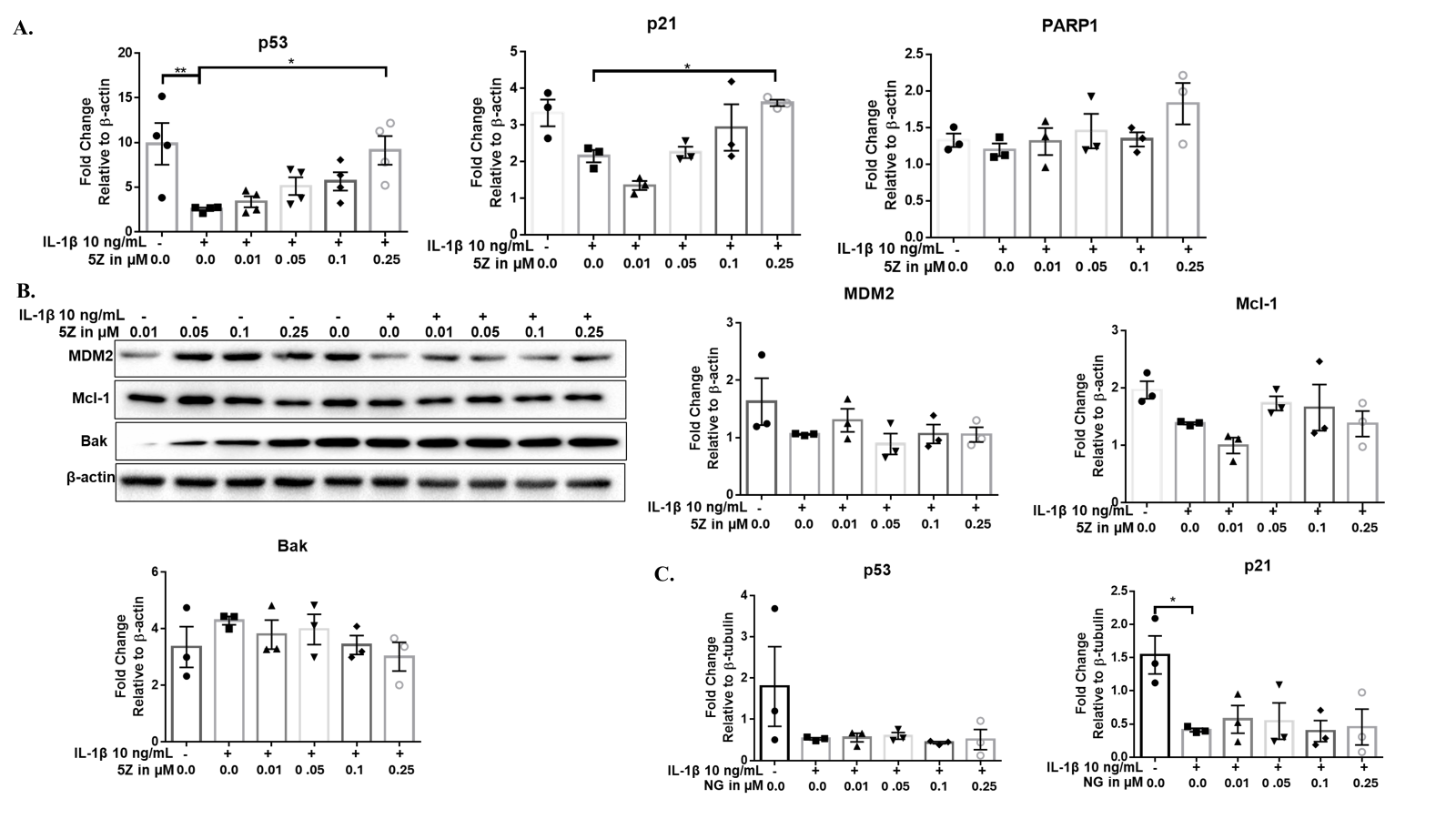


**Fig. S5: A.** Densitometric analysis of Western blots mentioned in Figure 4 B. **B.** JIASFs were pretreated with 5Z followed by IL-1β stimulation for 24 h, and the expression of MDM2, Mcl-1, and Bak was measured. **C.** Densitometric analysis of Western blots mentioned in Figure 4C. The results were analyzed using One-way ANOVA followed by Dunnett’s test. *p<0.05, and **p<0.01 were considered statistically significant. The values were represented as mean ± SEM, compared with IL-1β treatment.


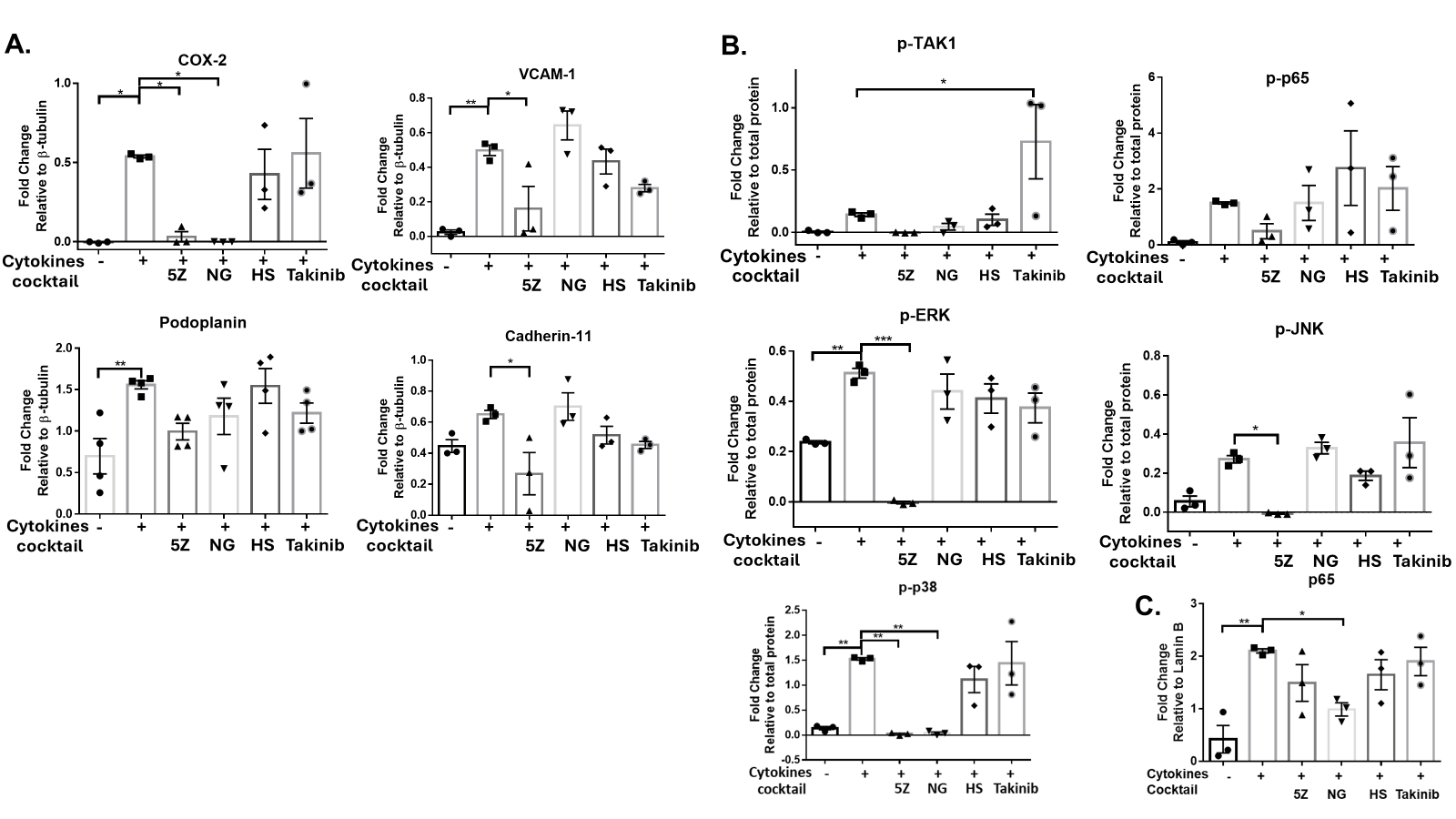


**Fig. S6: A, & B.** Densitometric analysis of Western blots mentioned in Figure 5A, F & G. The results were analyzed using One-way ANOVA followed by Dunnett’s test. *p<0.05, and **p<0.01 were considered statistically significant. The values were represented as mean ± SEM, compared with IL-1β treatment.

**Supplementary Table 1:** Clinical Information of the cell donors

|  | JIASFs Donor (n=8) | RASFs Donor (N=5) |
| --- | --- | --- |
| Age at sample collection  (Median years, Interquartile range) | 10, 22.25 | 61, 2 |
| Sex (% Female) | 62.5% | 100% |
| Types of JIA (n, %) | | |
| Oligo | 5, 62.5% | - |
| Unknown | 3, 37.5% | - |
| Ethnicity (n, %) | | |
| Non-Hispanic | 2, 25% | - |
| Undisclosed | 6, 75% | - |
| Self-reported Race (n, %) | | |
| White | 4, 50% | 1, 20% |
| Unknown | 4, 50% | 4, 80% |

**References**

1. Brescia, A.C., et al., *The role of transforming growth factor beta signaling in fibroblast-like synoviocytes from patients with oligoarticular juvenile idiopathic arthritis: dysregulation of transforming growth factor beta signaling, including overexpression of bone morphogenetic protein 4, may lead to a chondrocyte phenotype and may contribute to bony hypertrophy.* Arthritis Rheumatol, 2014. **66**(5): p. 1352–62.

2. Pelassa, S., et al., *Characterization of fibroblast-like synoviocytes from the synovial fluid of patients affected by juvenile idiopathic arthritis.* Rheumatology (Oxford), 2025.

3. Ahmed, S., A. Pakozdi, and A.E. Koch, *Regulation of interleukin-1beta-induced chemokine production and matrix metalloproteinase 2 activation by epigallocatechin-3-gallate in rheumatoid arthritis synovial fibroblasts.* Arthritis Rheum, 2006. **54**(8): p. 2393–401.

4. Fechtner, S., et al., *Cannabinoid Receptor 2 Agonist JWH-015 Inhibits Interleukin-1beta-Induced Inflammation in Rheumatoid Arthritis Synovial Fibroblasts and in Adjuvant Induced Arthritis Rat via Glucocorticoid Receptor.* Front Immunol, 2019. **10**: p. 1027.

5. Siegel, R.J., et al., *Extracellular sulfatase-2 is overexpressed in rheumatoid arthritis and mediates the TNF-alpha-induced inflammatory activation of synovial fibroblasts.* Cell Mol Immunol, 2022. **19**(10): p. 1185–1195.
